# One-Year Mortality Associations in Hemodialysis Patients after Traumatic Brain Injury—An Eight-Year Population-Based Study

**DOI:** 10.1371/journal.pone.0093956

**Published:** 2014-04-08

**Authors:** Jen-Chieh Liao, Chung-Han Ho, Fu-Wen Liang, Jhi-Joung Wang, Kao-Chang Lin, Chung-Ching Chio, Jinn-Rung Kuo

**Affiliations:** 1 Department of Neurosurgery, Chi-Mei Medical Center, Tainan, Taiwan; 2 Department of Medical Research, Chi-Mei Medical Center, Tainan, Taiwan; 3 Institute of Public Health, College of Medicine, National Cheng Kung University, Tainan, Taiwan; 4 Department of Biotechnology, Southern Taiwan University of Science and Technology, Tainan, Taiwan; 5 Department of Hospital and Health Care Administration, Chia Nan University of Pharmacy and Science, Tainan, Taiwan; Imperial College London, Chelsea & Westminster Hospital, United Kingdom

## Abstract

**Purpose:**

This study aimed to investigate the one-year mortality associations in hemodialysis patients who underwent neurosurgical intervention after traumatic brain injury (TBI) using a nationwide database in Taiwan.

**Materials and Methods:**

An age- and gender-matched longitudinal cohort study of 4416 subjects, 1104 TBI patients with end-stage renal disease (ESRD) and 3312 TBI patients without ESRD, was conducted using the National Health Insurance Research Database in Taiwan between January 2000 and December 2007. The demographic characteristics, length of stay (LOS), length of ICU stay, length of ventilation (LOV), and tracheostomy were collected and analyzed. The co-morbidities of hypertension (HTN), diabetes mellitus (DM), myocardial infarction (MI), stroke, and heart failure (HF) were also evaluated.

**Results:**

TBI patients with ESRD presented a shorter LOS, a longer length of ICU stay and LOV, and a higher percentage of comorbidities compared with those without ESRD. TBI patients with ESRD displayed a stable trend of one-year mortality rate, 75.82% to 76.79%, from 2000–2007. For TBI patients with ESRD, the median survival time was 0.86 months, and pre-existing stroke was a significant risk factor of mortality (HR: 1.29, 95% C.I.: 1.08–1.55). Pre-existing DM (HR: 1.35, 95% C.I.: 1.12–1.63) and MI (HR: 1.61, 95% C.I.: 1.07–2.42) effect on the mortality in ESRD patients who underwent TBI surgical intervention in the younger (age<65) and older (age≥65) population, respectively. In addition, the length of ICU stay and tracheostomy may provide important information to predict the mortality risk.

**Conclusions:**

This is the first report indicating an increased risk of one-year mortality among TBI patients with a pre-existing ERSD insult. Comorbidities were more common in TBI patients with ESRD. Physicians should pay more attention to TBI patients with ESRD based on the status of age, comorbidities, length of ICU stay, and tracheostomy to improve their survival.

## Introduction

The population of patients with end-stage renal disease (ESRD) requiring dialysis is progressively growing, and the mortality rate of this group is much higher than that of the general population in the USA in recent decades[1]. In Taiwan, cases of ESRD requiring hemodialysis also have increased considerably over the past few decades [2], [3]. The country has had the greatest incidence and the second greatest prevalence of ESRD since 2000, according to an international comparison based on data from the US Renal Data System [4], [5].

Patients who have pre-existing ESRD and are admitted to the intensive care unit (ICU) with different etiologies, such as medical [6], [7] or surgical problems [8], [9], have a higher mortality than patients without ESRD. The data also indicate that ESRD patients have a greater number of comorbidities, such as diabetes, stroke, hypertension, and heart disease, than non-ESRD patients admitted to the ICU [10]. Thus, these comorbidities are often one of the risk factors for mortality [11].

According to the Centers for Disease Control and Prevention, the annual incidence of traumatic brain injury (TBI) in the United States is ∼1.7 million [12]. Incidence rates of 103 and 344 per 100 000 people have been reported in the United States [13] and Taiwan [14], respectively. At present, TBI is a major cause of death and disability in humans and remains a critical public health challenge. Cases of ESRD requiring hemodialysis have increased in Taiwan. Therefore, neurosurgeons can expect to see more dialysis patients presenting with TBI in daily practice.

In central nervous system insults, several studies have demonstrated that the mortality rate after stroke is higher in ESRD patients than in the general population [15]–[18]. However, the long-term prognosis, the mortality rate, and possible risk factors in TBI patients with ESRD remain unclear. Therefore, the aim of this study was to evaluate the one-year mortality risk factors for TBI patients with ESRD using data from the nationwide database of the National Health Insurance (NHI) Program in Taiwan (2000–2007). We believe that awareness of the possibility of developing mortality post-TBI can improve not only one's understanding of the sequelae of brain injury but also the patient treatment and prevention protocol.

## Materials and Methods

### Data source

In this study, the inpatient medical claims data between January 1997 and December 2008 were retrieved from the Taiwan's National Health Insurance Research Database (NHIRD). NHIRD was established by the Taiwan's National Health Research Institute to enhance medical research. The NHIRD have has been used extensively in various published studies[19]. The database comprises detailed information about clinical visits for each insured subject, including diagnostic codes according to the clinical modification of the International Classification of Diseases, Ninth Revision, Clinical Modification (ICD-9CM) code. The confidentiality of individuals was protected using encrypted personal identification to avoid the possibility of the ethical violations related to the data. Exemption was obtained from the institutional review board of Chi Mei Medical Center.

### Patient selection and definition

The inpatient medical claims data during 2000–2007 was analyzed in this study. TBI patients were defined as those claiming medical expenditure applications for TBI surgery. The patients with ESRD were identified using the catastrophic illness certification records, as patients who underwent hemodialysis for more than three months could apply for a catastrophic illness card. A total of 74226 TBI patients received surgery, and of those, 1104 patients with ESRD were selected as the study sample. We then randomly selected 3312 TBI patients without ESRD (three for every patient with TBI) who were matched with the study cohort in terms of age and gender. The disease records for these patients were consistent with the format of ICD-9CM code. The co-morbidities of hypertension (HTN), diabetes mellitus (DM), myocardial infarction (MI), stroke, and heart failure (HF) were based on the records from one year before the date of TBI diagnosis. HTN was coded by hypertensive disease (ICD-9CM: 401-405), hypertensive encephalopathy (ICD-9CM: 437.2), and hypertensive retinopathy (ICD-9CM: 362.11). The DM was coded as follows: diabetes mellitus (ICD-9CM: 250), polyneuropathy in diabetes (ICD-9CM: 357.2), diabetic retinopathy (ICD-9CM: 362.0), and diabetic cataract (ICD-9CM: 366.41). MI was coded by acute myocardial infarction (ICD-9CM: 410) and old myocardial infarction (ICD-9CM: 412). Stroke was coded by cerebrovascular disease (ICD-9CM: 430-438). HF was coded by heart failure (ICD-9CM: 428), rheumatic heart failure (ICD-9CM: 398.91), acute myocarditis (ICD-9CM: 422), cardiomyopathy (ICD-9CM: 425), hypertensive heart disease with heart failure (ICD-9CM: 402.01, 402.11, and 402.91), and hypertensive heart and chronic kidney disease with heart failure (ICD-9CM: 404.01, 404.03, 404.11, 404.13, 404.91, and 404.93).

### Measurements

The main event in this study was one-year mortality as TBI patients with ESRD had short survival after surgery. Survival time was calculated from the date of TBI surgery to the date of death or to one year after admission. Demographic and clinical characteristics, including age, gender, level of urbanization[20], DM, HTN, MI, stroke, heart failure, length of stay (LOS), length of ICU stay, length of ventilation (LOV), and tracheostomy, were also used to estimate the risk of one-year mortality. For stratified analysis, patients were further classified into three groups according to their length of ICU stay: shorter group (first quartile), in-between group (first quartile to third quartile), and prolonged group (third quartile). This approach has been used on some previous studies for the distribution of ICU length of stay [21], [22].

### Statistical analysis

Comparisons between TBI patients with/without ESRD were performed using Pearson's chi-square test or Fisher's exact test for categorical variables and the Student's *t* test or Wilcoxon rank-sum test for continuous variables. The Kaplan-Meier plot was constructed to describe the proportion of patients who were still alive after one year, and the log-rank test was used to compare the mortality risk difference between two subgroups. The Cox proportional regression model was applied to estimate the hazards of mortality adjusted by potential confounding factors. To test the hypothesis that for TBI patients, the effect of pre-existing ESRD on mortality varied by age and the length of ICU stay, the model was also stratified by age and the length of ICU stay group to evaluate the potential risk factors. A *p*-value <0.05 was considered significant. All analyses were performed using Statistical Analysis System (SAS) statistical software (version 9.3; SAS Institute, Inc., Cary, NC). The Kaplan-Meier curves were plotted using STATA (version 12; Stata Corp., College Station, TX).

## Results

Table 1 shows the distribution of demographical and clinical variables for TBI patients with and without ESRD. A total of 4416 subjects (1104 TBI patients with ESRD and 3312 TBI patients without ESRD) were enrolled in this study. Of the TBI patients, most (64.13%) were younger than 65 years of age, and there were more males than females. The distribution of overall mortality indicated a significant difference between TBI patients with ESRD and those without ESRD. The median survival time was 0.86 (0.26–8.91) months for TBI patients with ESRD and 29.36 (4.18–59.57) months for those without ESRD. TBI patients with ESRD have a higher percentage of hospitalization deaths than do patients without ESRD. As most TBI patients with ESRD did not survive more than one year, the one-year mortality was used in this study to understand the potential risk factors. In addition, TBI patients with ESRD also presented a shorter LOS, a longer length of ICU days and LOV, and a higher percentage of comorbidities (HTN, DM, MI, stroke, and heart failure) compared with patients without ESRD.

**Table 1:** Demographic characteristics and clinical information of TBI surgery patients with and without pre-existing E SRD from 2000 to 2007 in Taiwan.

	With ESRD (N = 1104)	Without ESRD (N = 3312)	*p*-value*
**Age (mean±SD)**	60.05±12.29	60.05±12.29	0.9995
**Age (%)**			
**<65**	708(64.13)	2124(64.13)	1.0000
**> = 65**	396(35.87)	1188(35.87)	
**Gender (%)**			
**Male**	588(53.26)	1764(53.26)	1.0000
**Female**	516(46.74)	1548(46.74)	
**Level of Urbanization (%)**			
**1**	418(37.86)	1274(38.47)	0.8445
**2**	488(44.20)	1431(43.21)	
**> = 3**	198(17.93)	607(18.33)	
**Time to death, months**			
**Median(IQR)**	0.86(0.26–8.91)	29.36(4.18–59.57)	<.0001
**Death (%)**			
**Yes**	959(86.87)	1332(40.22)	<.0001
**No**	145(13.13)	1980(59.78)	
**Time to death at one year, month**			
**Median (IQR)**	0.86(0.26–8.91)	12(4.18–12.00)	<.0001
**Death at one year(%)**			
**Yes**	847(76.72)	986(29.77)	<.0001
**No**	257(23.28)	2326(70.23)	
**Hospitalization Death (%)**			
**Yes**	219(19.84)	227(6.85)	<.0001
**No**	885(80.16)	3085(93.15)	
**Length of Stay, days**			
**Median (IQR)**	14(6–28)	21(11–30)	<.0001
**Length of ICU, days**			
**Median (IQR)**	10(5–19)	8(4–16)	<.0001
**Length of ventilation, days**			
**Median (IQR)**	9(5–17)	6(3–14)	<.0001
**Tracheostomy (%)**			
**Yes**	213(19.29)	614(18.54)	0.5777
**No**	891(80.71)	2698(81.46)	
**Comorbidity**			
**Hypertension (%)**			
**Yes**	965(87.41)	1861(56.19)	<.0001
**No**	139(12.59)	1451(43.81)	
**Diabetes mellitus (%)**			
**Yes**	589(53.35)	895(27.02)	<.0001
**No**	515(46.65)	2417(72.98)	
**Myocardial infarction (%)**			
**Yes**	68(6.16)	91(2.75)	<.0001
**No**	1036(93.84)	3221(97.25)	
**Stroke (%)**			
**Yes**	889(80.53)	2168(65.46)	<.0001
**No**	215(19.47)	1144(34.54)	
**Heart failure (%)**			
**Yes**	196(17.75)	168(5.07)	<.0001
**No**	908(82.25)	3144(94.93)	

* ***p*** **-values were derived from the Student** ***t*** **test for continuous variables and chi-square test for categorical variables.**

**ESRD: end-stage renal disease; SD: standard deviation; IQR: interquartile range; ICU: Intensive Care Unit.**

**Figure 1:**
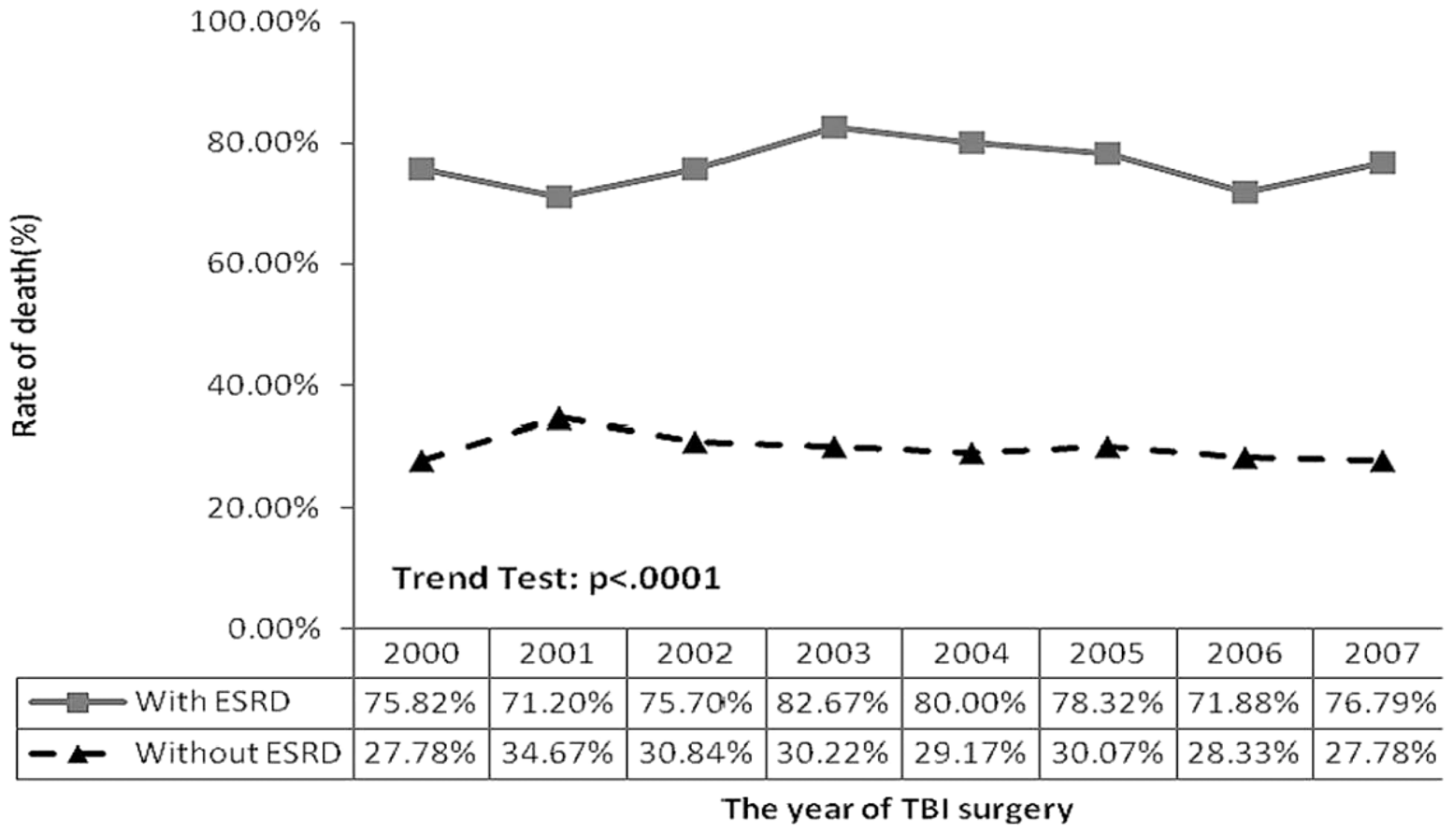
Trend of one-year mortality rate for TBI patients receiving surgery with and without pre-existing ESRD.

Figure 1 shows that both TBI patients with ESRD and without ESRD had a stable trend in one-year mortality rate during the follow-up period. The result of a trend test indicated there was a significant trend difference between TBI with ESRD patients and patients without ESRD (*p*<.0001).

**Figure 2:**
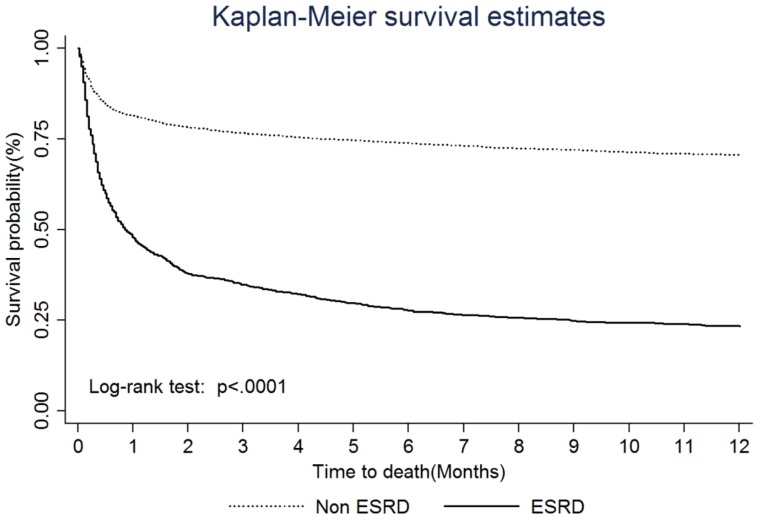
Kaplan-Meier survival curves for mortality of TBI surgery patients with and without pre-existing ESRD.

The Kaplan-Meier survival curves for TBI patients with and without ESRD are presented in Figure 2. There was a significant difference in survival between the two groups (*p*<.0001). In addition, after controlling for age, gender, and clinical characteristics, TBI patients with ESRD were more likely to die within one year (HR: 2.85, 95%C.I.: 2.56–3.17) compared with TBI patients without ESRD. Kaplan-Meier plots indicated a mortality rate of 52.36% at 1 month, 65.40% threat 3 months, 72.46% at 6 months, and 76.63% for one year.

Table 2 shows the hazard ratios of one-year mortality for TBI patients with ESRD. The hazard ratio (HR) for TBI patients indicates that an age ≥65 years (HR: 1.21, 95% C.I.: 1.05–1.39), stroke (HR: 1.29, 95% C.I.: 1.08–1.55), and prolonged use of a ventilator (HR: 1.06, 95% C.I.: 1.05–1.08) were associated with a significantly higher risk of mortality. However, a longer LOS (HR: 0.93, 95% C.I.: 0.92–0.94), longer ICU stays, and tracheostomy use were associated with a significantly lower risk of mortality.

**Table 2:** Univariate and multivariable Cox proportional hazards regression analysis for the mortality rate ratios of composite outcome among TBI patients with pre-existing ESRD.

	Crude Hazards (95% C.I.)	*p*-value	Adjusted Hazards* (95% C.I.)	*p*-value
**Age**				
**<65**	1.00(Ref.)		1.00(Ref.)	
**> = 65**	1.23(1.07–1.42)	0.0030	1.21(1.05–1.39)	0.0081
**Gender**				
**Female**	1.00(Ref.)		1.00(Ref.)	
**Male**	0.98(0.86–1.12)	0.7633	0.96(0.83–1.10)	0.5151
**Level of Urbanization**				
**> = 3**	1.00(Ref.)		1.00(Ref.)	
**1**	0.93(0.77–1.12)	0.4324	0.94(0.77–1.13)	0.4951
**2**	0.85(0.70–1.03)	0.0896	0.94(0.77–1.13)	0.4891
**Hypertension**				
**No**	1.00(Ref.)		1.00(Ref.)	
**Yes**	0.83(0.69–1.02)	0.0720	0.88(0.71–1.08)	0.2252
**Diabetes mellitus**				
**No**	1.00(Ref.)		1.00(Ref.)	
**Yes**	1.10(0.96–1.26)	0.1766	1.14(0.98–1.31)	0.0810
**Myocardial infarction**				
**No**	1.00(Ref.)		1.00(Ref.)	
**Yes**	1.02(0.77–1.35)	0.8970	1.04(0.78–1.39)	0.7708
**Stroke**				
**No**	1.00(Ref.)		1.00(Ref.)	
**Yes**	1.21(1.02–1.44)	0.0339	1.29(1.08–1.55)	0.0052
**Heart failure**				
**No**	1.00(Ref.)		1.00(Ref.)	
**Yes**	1.08(0.91–1.29)	0.3754	0.96(0.80–1.15)	0.6321
**Length of Stay, days**	0.95(0.94–0.96)	<.0001	0.93(0.92–0.94)	<.0001
**Length of ICU stay, days**				
**< = 5**	1.00(Ref.)		1.00(Ref.)	
**6∼19**	0.59(0.50–0.69)	<.0001	0.61(0.51–0.73)	<.0001
**>19**	0.40(0.33–0.48)	<.0001	0.52(0.38–0.72)	<.0001
**Length of ventilation, days**	0.99(0.98–0.99)	<.0001	1.06(1.05–1.08)	<.0001
**Tracheostomy**				
**No**	1.00(Ref.)		1.00(Ref.)	
**Yes**	0.61(0.51–0.72)	<.0001	0.73(0.59–0.90)	0.0034

*The model was adjusted by the variables which were listed above.

**C.I.: confidence interval; Ref.: reference; ICU: Intensive Care Unit.**

**Figure 3:**
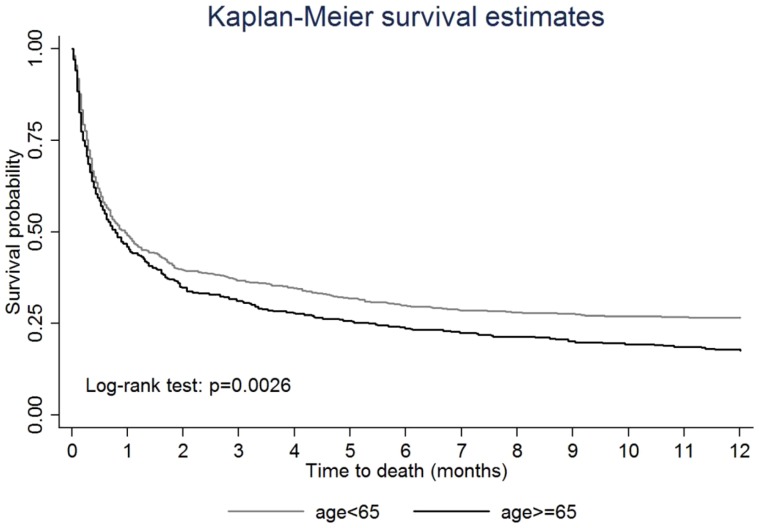
Kaplan-Meier survival curves for mortality of TBI patients with pre-existing ESRD, stratified by age.

In Figure 3, the Kaplan-Meier plots show that the risk of mortality for TBI patients with ESRD is significantly different between the <65 y/o and ≥65 y/o patients (*p* = 0.0026). However, at the beginning of the study period, the survival probability was similar between these two groups. About one month after surgery, TBI patients with ESRD who were ≥65 years old presented a higher risk of mortality than did the younger patients.

When stratified by age, DM (HR: 1.35, 95% C.I.: 1.12–1.63) and LOV (HR: 1.07, 95% C.I.: 1.05–1.09) were significant risk factors for TBI patients with ESRD who were younger than 65 y/o (Table 3). For older patients (age ≥65 y/o), MI (HR: 1.61, 95% C.I.: 1.07–2.42) and LOV (HR: 1.05, 95% C.I.: 1.03–1.07) were significant risk factors. LOS and length of ICU stay were the protective factors of mortality regardless of age. Although tracheostomy was a protective factor, it was significant for young patients and borderline significant for older patients.

**Table 3:** Mortality rate ratios of composite outcome among TBI patients with pre-existing ESRD, stratified by age.

	Age<65	*p*-value	Age> = 65	*p*-value
	Adjusted Hazards* (95% C.I.)		Adjusted Hazards* (95% C.I.)	
**Gender**				
**Female**	1.00(Ref.)		1.00(Ref.)	
**Male**	0.98(0.83–1.17)	0.8487	0.89(0.72–1.11)	0.3173
**Level of Urbanization (%)**				
**> = 3**	1.00(Ref.)		1.00(Ref.)	
**1**	1.00(0.78–1.29)	0.9839	0.94(0.69–1.29)	0.7139
**2**	0.90(0.71–1.16)	0.4261	1.04(0.77–1.42)	0.7887
**Hypertension**				
**No**	1.00(Ref.)		1.00(Ref.)	
**Yes**	0.93(0.72–1.20)	0.5688	0.86(0.60–1.23)	0.3993
**Diabetes mellitus**				
**No**	1.00(Ref.)		1.00(Ref.)	
**Yes**	1.35(1.12–1.63)	0.0016	0.85(0.67–1.07)	0.1646
**Myocardial infarction**				
**No**	1.00(Ref.)		1.00(Ref.)	
**Yes**	0.74(0.48–1.13)	0.1591	1.61(1.07–2.42)	0.0211
**Stroke**				
**No**	1.00(Ref.)		1.00(Ref.)	
**Yes**	1.22(0.95–1.57)	0.1118	1.27(0.98–1.66)	0.0741
**Heart failure**				
**No**	1.00(Ref.)		1.00(Ref.)	
**Yes**	1.03(0.80–1.31)	0.8456	0.88(0.67–1.17)	0.3839
**Length of Stay, days**	0.92(0.91–0.93)	<.0001	0.95(0.93–0.96)	<.0001
**Length of ICU group**				
**< = 5**	1.00(Ref.)		1.00(Ref.)	
**6∼19**	0.69(0.54–0.88)	0.0029	0.53(0.40–0.71)	<.0001
**>19**	0.61(0.40–0.93)	0.0203	0.43(0.27–0.71)	0.0008
**Length of ventilation, days**	1.07(1.05–1.09)	<.0001	1.05(1.03–1.07)	<.0001
**Tracheostomy**				
**No**	1.00(Ref.)		1.00(Ref.)	
**Yes**	0.73(0.56–0.96)	0.0230	0.72(0.51–1.01)	0.0600

*The model was adjusted by the variables which were listed above.

**C.I.: confidence interval; Ref.: reference; ICU: Intensive Care Unit.**

In addition, stratified analysis by the length of ICU stay was presented in the Table 4. LOS is a significant protective factor for all subgroups. For shorter length of ICU stay, older TBI patients with ESRD had a 30% increased risk of one-year mortality compared to younger TBI patients. For patients with prolonged length of ICU stays, LOV days (HR: 1.03, 95% C.I.: 1.02–1.04) was the significant risk factor, but the comorbidity of HTN and LOS were the protective factors of one-year mortality. For the in-between group, stroke (HR: 1.35, 95% C.I.: 1.03–1.77) and LOV days (HR: 1.04, 95% C.I.: 1.02–1.07) were associated with increased risk of mortality whereas tracheostomy was associated with lower risk (HR: 0.68, 95% C.I.: 0.49–0.93).

**Table 4:** Mortality rate ratios of composite outcome among TBI patients with pre-existing ESRD, stratified by the length of ICU stay.

Adjusted Hazards* (95% C.I.)	Length of ICU stay, < = 5 days (N = 337)	*p*-value	Length of ICU stay, 6∼19 days (N = 501)	*p*-value	Length of ICU stay, >19 days (N = 266)	*p*-value
**Age**						
**<65**	1.00(Ref.)		1.00(Ref.)		1.00(Ref.)	
**> = 65**	1.30(1.01–1.67)	0.0409	1.07(0.86–1.33)	0.5660	1.20(0.89–1.62)	0.2260
**Gender**						
**Female**	1.00(Ref.)		1.00(Ref.)		1.00(Ref.)	
**Male**	1.09(0.85–1.39)	0.5156	0.97(0.79–1.18)	0.7306	0.79(0.58–1.06)	0.1142
**Level of urbanization**						
**> = 3**	1.00(Ref.)		1.00(Ref.)		1.00(Ref.)	
**1**	0.86(0.62–1.21)	0.3940	0.98(0.74–1.30)	0.8653	1.06(0.67–1.68)	0.7967
**2**	0.97(0.69–1.35)	0.8420	0.88(0.66–1.16)	0.3610	1.07(0.68–1.68)	0.7743
**Hypertension**						
**No**	1.00(Ref.)		1.00(Ref.)		1.00(Ref.)	
**Yes**	0.98(0.69–1.39)	0.9229	0.79(0.58–1.09)	0.1482	0.53(0.33–0.84)	0.0077
**Diabetes mellitus**						
**No**	1.00(Ref.)		1.00(Ref.)		1.00(Ref.)	
**Yes**	1.20(0.94–1.53)	0.1523	1.23(0.98–1.53)	0.0709	1.09(0.80–1.47)	0.5964
**Myocardial infarction**						
**No**	1.00(Ref.)		1.00(Ref.)		1.00(Ref.)	
**Yes**	1.18(0.74–1.89)	0.4777	0.77(0.45–1.30)	0.3224	1.17(0.67–2.04)	0.5765
**Stroke**						
**No**	1.00(Ref.)		1.00(Ref.)		1.00(Ref.)	
**Yes**	1.11(0.81–1.52)	0.5218	1.35(1.03–1.77)	0.0322	1.05(0.71–1.55)	0.8044
**Heart failure**						
**No**	1.00(Ref.)		1.00(Ref.)		1.00(Ref.)	
**Yes**	0.91(0.66–1.25)	0.5508	0.91(0.67–1.22)	0.5156	1.34(0.91–1.98)	0.1339
**Length of Stay, days**	0.84(0.81–0.88)	<.0001	0.92(0.91–0.94)	<.0001	0.97(0.95–0.98)	<.0001
**Length of ventilation, days**	1.06(0.99–1.13)	0.0880	1.04(1.02–1.07)	<.0001	1.03(1.02–1.04)	<.0001
**Tracheostomy**						
**No**	1.00(Ref.)		1.00(Ref.)		1.00(Ref.)	
**Yes**	0.30(0.04–2.15)	0.2282	0.68(0.49–0.93)	0.0169	0.82(0.61–1.12)	0.2207

*The model was adjusted by the variables which were listed above.

**C.I.: confidence interval; Ref.: reference; ICU: Intensive Care Unit.**

## Discussion

To the best of our knowledge, this is the first study to report one-year mortality associations in ESRD patients after TBI using population-based administrative data. The main findings of our study were that (1) TBI patients with ESRD had more co-morbidity, higher one-year mortality, and longer length of ventilator use compared with non-ESRD TBI patients; (2) age, comorbidity of stroke, and LOV were the risk factors of mortality for TBI patients with ESRD. However, LOS, length of ICU stay, and tracheostomy were protective factors for the same patients; (3) DM was a mortality predictor for ESRD patients whose age <65 y/o, and MI was a mortality predictor for those patients ≥65 years of age; and (4) the risk factors for mortality among TBI patients with ESRD varied by length of ICU stays. Given that the NHIRD covers nearly 99% of inpatient and outpatient medical benefit claims for the 23 million residents in Taiwan, the data here closely approximate the true distribution of ESRD in TBI patients. For patients belonging to one of the high-risk groups, this information is critical to clinicians when they perform the TBI surgery and when they explain the prognosis to the patient's family.

### High mortality rate was observed in TBI patients with ESRD who underwent surgical intervention compared with patients without ESRD

Several studies have reported that acute kidney injury may develop due to renal ischemia following hypoperfusion as a complication of traumatic brain injury [23], [24]. However, pre-existing ESRD effects on TBI patients were not evaluated. Our finding indicated that TBI patients with ESRD had a higher percentage of hospitalization and one-year mortality, ICU days, length of ventilation, and comorbidities than patients without ESRD. The hazard ratio of one-year mortality, which was adjusted by age, gender, and clinical variables for TBI patients between those with ESRD and those without ESRD, is 2.85 (95% C.I.: 2.56–3.17). The results provide evidence supporting a new concept that pre-existing ESRD is a risk factor of mortality after TBI surgery. In addition, ESRD is associated with increased mortality among patients who are admitted to the ICU, and this effect is mostly a result of comorbidity [10], [25]. Our results are also consistent with previous reports that ESRD patients have a greater incidence of comorbidities such as diabetes, stroke, hypertension, heart disease than patients without ESRD. This may support that the conclusion that ESRD patients use a significantly greater amount of resources than do non-ESRD patients [26], [27]. Therefore, the investigation of the impact of neurosurgical interventions on healthcare costs and economic burden in dialysis patients, who are a growing population with high co-morbidity, is a very important issue for the future.

In addition, our results are consistent with those of Chapman et al. [28], who reported that the majority of deaths occurred within the first month of ICU admission, depending on the medical etiology. In the current study, we found that the majority of deaths occurred at 0.86 months (IQR: 0.26–8.91), with a one-month post-ICU admission mortality rate of 52.36% (49.44%–55.33%). In our study, the one-year mortality rate of 76.63% (74.10%–79.08%) was higher than the previously reported 40% to 65% for critically ill ESRD patients [7], [29], [30]. Our results are also consistent with those of Rhodes et al. and Dhanani et al. who indicated that patients who had pre-existing ESRD after major surgery had a higher mortality than patients without ESRD [8], [31]. Furthermore, in the current study, the one-year mortality trend test from 2000 to 2007 between TBI patients with ESRD and TBI patients without ESRD revealed significant differences (76.63% vs. 29.77%). These results imply that despite aggressive surgical intervention, the one-year mortality remained high and stable (76.63%). Thus, patients with pre-existing ERSD who suffer TBI and require neurosurgical intervention should be considered to have a serious and critical condition. This important information can be useful to surgeons, families, intensivists and nephrologists when considering surgical intervention and defining goals for treatment.

Studies have shown that hypertension, DM, MI, stroke, and heart failure are associated with higher mortality in TBI patients [32], [33]. However, how these comorbidities affect the prognosis in TBI patients with ESRD is still unknown. This is the first study to examine the effect of comorbidities on mortality among TBI with ESRD after controlling for potential risk factors. With the insult of ESRD, hypertension, DM, MI, and heart failure are not significantly associated with higher mortality for TBI patients.

### Pre-existing stroke is a risk factor of mortality for ESRD patients with TBI who underwent surgical intervention

The overall mortality of spontaneous intracerebral hemorrhage in ESRD patients was 61% to 71.4%, and the mortality rate after stroke was 46.6% at one month and 64.3% at one year [17], [18]. Thus, stroke is an important risk factor of mortality for ESRD patients. In addition, the study by Thompson and colleagues demonstrated that the cerebrovascular disease had high association with mortality [32]. Our findings also indicate that pre-existing stroke is a significant risk factor for ESRD patients with TBI who underwent surgical intervention. Among these patients, those with preexisting stroke displayed a 29% increase in mortality risk compared with those without preexisting stroke (HR: 1.29, 95% C.I.: 1.08–1.55).

However, we believe that the effects of different types of intracranial hemorrhage, such as epidural hemorrhage, subdural hemorrhage, and intracerebral hemorrhage, on TBI mortality rate deserve to be investigated in the future. Therefore, we recommend the link between pre-existing stroke and TBI, effects of severity, subtypes, and external causes of TBI should be examined in future studies.

### Pre-existing DM and MI effects on the mortality in ESRD patients who underwent TBI surgical intervention in the younger (age<65) and older (age≥65) population, respectively

Age has long been recognized as an independent adverse outcome predictor following TBI in various studies. Older age is associated with poorer outcome in patients with TBI [34]–[36]. Consistent with previous studies, we found the long-term mortality rate starting from one month (60.45% vs. 65.66%) to one year (73.45% vs 82.32%) after TBI in ESRD patients was significantly different between patients <65 y/o and patients ≥65 y/o. We also found significantly elevated one-year mortality rates in the older dialysis patients compared with the younger patients after TBI surgery (HR: 1.21, 95% C.I.: 1.05–1.39). These findings are consistent with the US Renal Data Service annual report that indicates that among dialysis patients age 65 and older, the mortality is twice as high as that in the general population [37]. Given that societal aging may increase the number of surgical cases of ESRD with TBI and that the older population presents a high mortality risk, neurosurgeons should be more cautious when performing surgical intervention in older ESRD patients if TBI has occurred.

Currently, diabetic nephropathy is the leading cause of ESRD in Germany and Taiwan [27], [38]. Many studies have reported an association between pre-existing DM at the initiation of dialysis and a poor outcome in ESRD patients undergoing dialysis [38], [39]. We further elucidated that for TBI patients with ESRD, pre-existing DM is a risk factor of one-year mortality (HR: 1.35, 95% C.I.: 1.12–1.63) for patients <65 years of age. One possible hypothesis is that younger ESRD patients have a longer follow-up related to DM, so a poorer health condition leads to a higher mortality risk for those who undergo surgical intervention. We believe that the management of TBI patients with ESRD derived from DM will represent a serious problem in the future.

In addition, pre-existing MI was associated with a significantly increased risk of mortality (HR: 1.61, 95% CI: 1.07–2.42) in the older population (age≥65). The reason for this result might be that pre-existing MI in older patients is a key factor to determine mortality compared with other comorbidities. Because we evaluated neither the time interval since MI nor the severity of MI, which would influence cardiac function and mortality, we believe this information encourages us to pay more attention to these new high-risk groups during the decision-making process for surgical intervention.

### Length of ICU stay could be a mortality predicator and a surrogate of disease severity for ESRD patients who underwent TBI surgery

Previous studies found that the TBI patients with prolonged ICU stays have greater disease severity or poor functional outcome according to the different scores [40]–[44]. Since the TBI patients were unstable after TBI surgery at the beginning of ICU stay, and longer ICU lengths of stay might be associated with greater disease severity, examining the risk factors for mortality by different lengths of ICU stay among TBI patients is meaningful. Results of stratified analysis indicated that patients with different lengths of ICU stays have different risk factors for mortality: for patients with short ICU stays, age was an independent risk factor for mortality, and it was similar as former studies that age is the most frequently documented risk factor [45], [46]; for those with prolonged ICU stays, HTN was a protective factor, and this “ reverse epidemiology” had also been reported on the some studies for ESRD patients [47], [48]; for in-between groups, stroke and LOV were associated with increased risk of death, but tracheostomy was associated with lower risk.

One reason for tracheostomy need may be related to general clinical practices and the general consensus of neurosurgeons that tracheostomy is not required for patients who have a critical condition and less opportunity to survive. The previous studies indicated that the benefits of tracheostomy have been demonstrated for critically ill patients, especially for patients with limited neurological recovery, without spontaneous ventilation, excessive secretion, and long-term functional outcome [49]–[51]. However, the association between the tracheostomy and mortality is still unclear. A meta-analysis review indicated that the tracheostomy could reduce the risk of mortality, but it is no significant [21]. Only one study demonstrated that tracheostomy could significantly reduce mortality [52]. In our study, the effect of tracheostomy on mortality presented the protective information in the different ICU stays, only the in-between ICU stays group of TBI patients with ESRD who received tracheostomy displayed a 32% reduction in the one-year mortality rate compared with those who did not receive tracheostomy (HR: 0.68, 95% C.I.: 0.49–0.93). Since trauma patients are unstable at short length of ICU stay, tracheostomy may not be applied according the guideline[49]–[51]. Thus, it could not be significant for protecting the mortality. Considering of the severity of TBI, TBI patients with ESRD within prolonged ICU days may have the poor functional outcomes. The non-significant protective effect on mortality is consistency as previous studies [21], [22], [41]. Therefore, the tracheostomy may still have some benefit for some restricted patients. This information can be provided to the families of patients if they have doubts about the tracheotomy procedure in ESRD patients who underwent TBI surgery.

## Limitations

There are several limitations in our study. First, the diagnoses, including comorbidities, all relied on the claims data and ICD-9-CM diagnosis codes; thus, there may be some disease misclassification. Second, we were unable to take into account the illness severity scores of TBI and ESRD because the data were unavailable. Third, the data concerning the onset, duration of tracheostomy, and severity of DM, MI, and stroke were also unavailable for evaluation.

## Conclusions

TBI patients with ESRD have more co-morbidity and higher one-year mortality than with non-ESRD TBI patients. Age, history of comorbidities, length of ICU stay, and tracheostomy are associated with higher mortality in TBI patients with ESRD. When performing TBI surgery or explaining the prognosis to the families of patients, physicians should keep these factors in mind to the aforementioned high-risk groups.
